# Insights into *Vibrio cholerae* Intestinal Colonization from Monitoring Fluorescently Labeled Bacteria

**DOI:** 10.1371/journal.ppat.1004405

**Published:** 2014-10-02

**Authors:** Yves A. Millet, David Alvarez, Simon Ringgaard, Ulrich H. von Andrian, Brigid M. Davis, Matthew K. Waldor

**Affiliations:** 1 Division of Infectious Diseases, Brigham and Women's Hospital, Boston, Massachusetts, United States of America; 2 Department of Microbiology and Immunobiology, Harvard Medical School, Boston, Massachusetts, United States of America; 3 Howard Hughes Medical Institute, Boston, Massachusetts, United States of America; University of Texas, San Antonio, United States of America

## Abstract

*Vibrio cholerae*, the agent of cholera, is a motile non-invasive pathogen that colonizes the small intestine (SI). Most of our knowledge of the processes required for *V. cholerae* intestinal colonization is derived from enumeration of wt and mutant *V. cholerae* recovered from orogastrically infected infant mice. There is limited knowledge of the distribution of *V. cholerae* within the SI, particularly its localization along the villous axis, or of the bacterial and host factors that account for this distribution. Here, using confocal and intravital two-photon microscopy to monitor the localization of fluorescently tagged *V. cholerae* strains, we uncovered unexpected and previously unrecognized features of *V. cholerae* intestinal colonization. Direct visualization of the pathogen within the intestine revealed that the majority of *V. cholerae* microcolonies attached to the intestinal epithelium arise from single cells, and that there are notable regiospecific aspects to *V. cholerae* localization and factors required for colonization. In the proximal SI, *V. cholerae* reside exclusively within the developing intestinal crypts, but they are not restricted to the crypts in the more distal SI. Unexpectedly, *V. cholerae* motility proved to be a regiospecific colonization factor that is critical for colonization of the proximal, but not the distal, SI. Furthermore, neither motility nor chemotaxis were required for proper *V. cholerae* distribution along the villous axis or in crypts, suggesting that yet undefined processes enable the pathogen to find its niches outside the intestinal lumen. Finally, our observations suggest that host mucins are a key factor limiting *V. cholerae* intestinal colonization, particularly in the proximal SI where there appears to be a more abundant mucus layer. Collectively, our findings demonstrate the potent capacity of direct pathogen visualization during infection to deepen our understanding of host pathogen interactions.

## Author Summary

*Vibrio cholerae* is a highly motile bacterium that causes the diarrheal disease cholera. Despite our extensive knowledge of the genes and processes that enable this non-invasive pathogen to colonize the small intestine, there is limited knowledge of the pathogen's fine localization within the intestine. Here, we used fluorescence microscopy-based techniques to directly monitor where and how fluorescent *V. cholerae* localize along intestinal villi in infected infant mice. This approach enabled us to uncover previously unappreciated features of *V. cholerae* intestinal colonization. We found that most *V. cholerae* microcolonies appear to arise from single cells attached to the epithelium. Unexpectedly, we observed considerable differences between *V. cholerae* fine localization in different parts of the small intestine and found that *V. cholerae* motility exerts a regiospecific influence on colonization. The abundance of intestinal mucins appears to be an important factor explaining at least some of the regiospecific aspects of *V. cholerae* intestinal localization. Overall, our findings suggest that direct observation of fluorescent pathogens during infection, coupled with genetic and/or pharmacologic manipulations of pathogen and host processes, adds a valuable depth to understanding of host-pathogen interactions.

## Introduction

Cholera, a severe and potentially fatal diarrheal disease, is caused by ingestion of food or water contaminated with the highly motile gram-negative rod *Vibrio cholerae*. Although the disease has been recognized for centuries, cholera still causes significant morbidity and mortality in several parts of the developing world, and it is an ongoing threat to public health in regions where access to clean water and adequate sanitation is limited [1]. For example, since the accidental introduction of *V. cholerae* to Haiti following a 2010 earthquake, cholera has already sickened ∼700,000 and killed more than 8,500 (http://www.mspp.gouv.ht/). *V. cholerae* is a non-invasive pathogen that colonizes the mucosal surface of the small intestine (SI). The majority of *V. cholerae*, including strains of the El Tor biotype within the O1 serogroup – the cause of the ongoing seventh pandemic of cholera - do not induce damage to host tissue; instead, mortality is principally due to the extreme dehydration that ensues from disease-associated diarrhea.

Analyses of *V. cholerae* infections in several animal models of disease, as well as in human volunteers, have enabled identification of numerous factors that contribute to bacterial colonization and disease. A key element is *V. cholerae's* production of cholera toxin, an ADP-ribosylating toxin that accounts for cholera's hallmark secretory diarrhea [2]. The toxin is not directly required for bacterial colonization of mammalian hosts [3]; however, due to the profuse diarrhea it induces, the toxin is thought to promote bacterial dissemination to new hosts. Cholera pathogenesis is also dependent upon *V. cholerae's* production of a type IV pilus, TCP, whose expression is co-regulated with cholera toxin [4], [5]. TCP is essential for *V. cholerae* to colonize the SI; it promotes bacterial aggregation and microcolony formation, and may also facilitate *V. cholerae's* adhesion to the mucosal surface and protect *V. cholerae* from antimicrobial agents in the intestine [6]. Additional genes and processes that are critical for *V. cholerae* survival and growth in vivo include LPS O-antigen, transport systems, such as RND efflux pumps [7], and metabolic processes, including biosynthesis of certain amino acids [8] [9] [10] (reviewed in [11]). Many of these have been identified in studies of suckling mice orogastrically infected with *V. cholerae*, a disease model that was developed more than 40 years ago.

Processes and apparati that modulate *V. cholerae* motility also influence intestinal colonization by this pathogen. Early studies showed that non-motile *V. cholerae* mutants had reduced virulence, and it was proposed that motility could enable the pathogen to penetrate the mucus barrier covering the epithelium [12]. More recently, targeted mutations that inactivate *V. cholerae's* single polar flagellum have also been shown to inhibit intestinal colonization [13]. Flagellum-based motility may enable the pathogen to reach preferred niches within the intestine; however, only its effect on net bacterial accumulation within the intestine has been investigated. Flagellum-based motility is also necessary for *V. cholerae* chemotaxis, but chemotaxis and motility mutants have distinct phenotypes in vivo. Of *V. cholerae's* 3 clusters of genes that encode chemotaxis-related proteins, only genes in cluster 2 have been found to be required for chemotaxis in vitro [14] [15]. Unexpectedly, cluster 2 mutants exhibit enhanced intestinal colonization in infant mice, particularly but not exclusively in the proximal intestine [16] [13] [17]. In particular, hypercolonization is associated with non-chemotactic *V. cholerae* mutants that exhibit counter clockwise-biased flagellar rotation, which results in longer stretches of smooth swimming and greater net movement, while mutants with clockwise-biased flagellar rotation reverse their swimming direction more often and exhibit attenuated colonization [17]. It has been proposed that chemotaxis facilitates movement toward the pathogen's preferential site of colonization in the distal half of the SI [17], [18]. The niches colonized by chemotaxis-deficient strains have not been identified.

Host factors and processes are also thought to modulate *V. cholerae's* capacity to colonize the SI, although there have been far fewer studies of these than of bacterial attributes. The acidic pH of the stomach is thought to kill most *V. cholerae* before the pathogen reaches the SI. Within the SI, mechanical and physical barriers include motility, which propels ingested and secreted material (e.g. mucus) toward the distal intestine, the mucus layer, which covers and protects the epithelial surface, and immune effectors (e.g. cryptidins), all of which are thought to limit *V. cholerae* colonization [19] [20]. The main component of the single layer of mucus that covers the small intestine is the mucin MUC-2, a large and highly glycosylated protein secreted by goblet cells [20]. The mucus layer is a highly viscous and complex structure, due in part to the disulphide crosslinks that form between mucin monomers [21]. Additional mucins that (unlike the mucus layer) are anchored to the epithelial cell membrane constitute the glycocalyx, another important protective barrier for the epithelium.

To date, most analyses of *V. cholerae* colonization and pathogenesis have not included analyses of the distribution of this pathogen within the SI or the bacterial and host factors that account for it. Enumeration of colony forming units (cfu) recoverable from different regions of the suckling mouse intestine has revealed that the proximal third of the SI harbors 40–100 fold less bacteria than the middle and distal regions [22]; however, this disparity has not been explained. Furthermore, with the exception of work monitoring fluorescently labeled *V. cholerae* in rabbit ligated ileal loops, which bypass the pathogen's ordinary route into the intestine [23], there is scant knowledge of how *V. cholerae* localizes along the villous axis in different regions of the SI. Here, we used confocal and two-photon microscopy to analyze the fine localization of fluorescent *V. cholerae* in different regions of the SI. Our observations suggest that most *V. cholerae* microcolonies arise from single cells attached to the epithelium. Unexpectedly, there are differences in *V. cholerae* localization in different regions of the SI. Notably, in the proximal SI, bacteria reside exclusively within the developing intestinal crypts. Furthermore, there are regiospecific requirements for motility in *V. cholerae* colonization; motility is critical for colonization of the proximal, but not the distal SI. Unexpectedly, neither motility nor chemotaxis were required for proper *V. cholerae* distribution along the villous axis, suggesting that yet undefined processes enable the pathogen to find its niches in the intervillous space. Additionally, our findings suggest that host mucins are a key inhibitor of *V. cholerae* colonization, particularly in the proximal SI.

## Results

### *V. cholerae* fine localization varies along the length of the small intestine

In order to visualize *V. cholerae* within intestinal tissue from infected infant mice, we orogastrically inoculated animals with fluorescent derivatives of C6706, a 7th pandemic El Tor O1 *V. cholerae* isolate. One strain (VcRed) constitutively expresses a codon-optimized gene encoding the red fluorescent protein tdTomato (tdT), while a comparable C6706 derivative constitutively produces GFPmut3 (VcGreen) [24]. The growth of VcRed and VcGreen was indistinguishable from that of C6706, both in LB cultures and in the small intestines (SI) of suckling mice, as assessed by competition assays (Figure S1). These data suggest that VcRed and VcGreen can be used as reliable reporters of *V. cholerae* localization during infection of infant mice.

**Figure 1:**
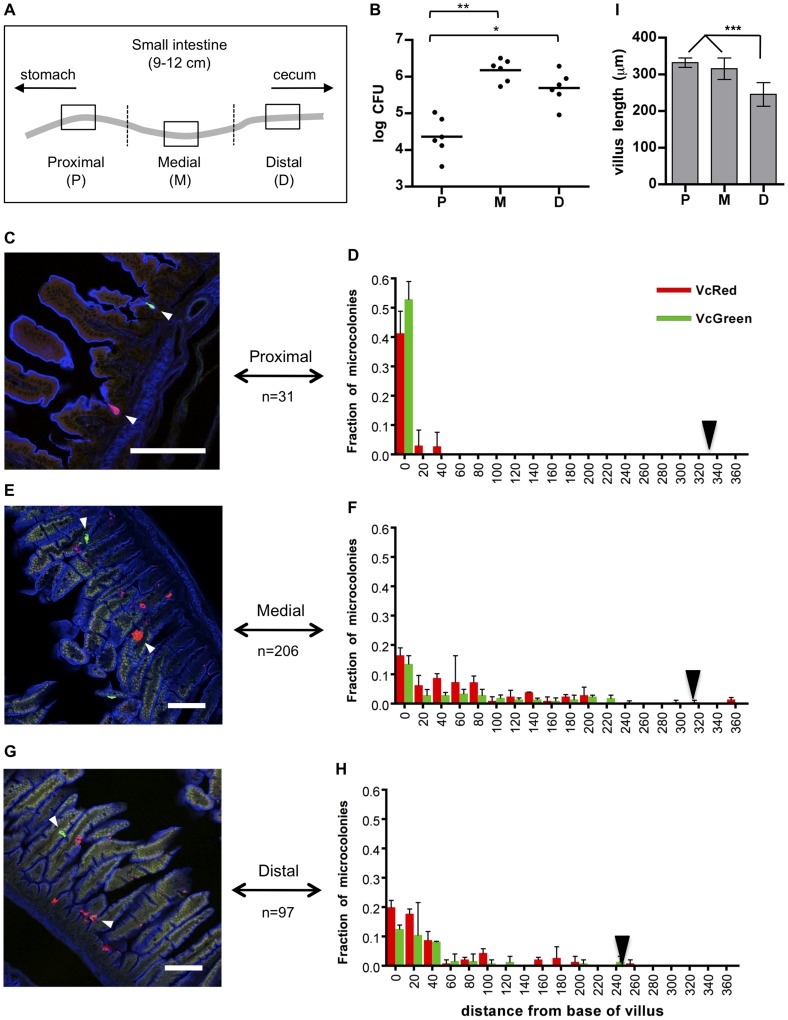
Distribution of fluorescently labeled *V. cholerae* in the infant mouse small intestine. (A) The small intestines of infant mice co-inoculated with VcRed and VcGreen were divided into three equal parts and the central 1 cm segments of the proximal (P), medial (M) and distal (D) parts were used for plating and microscopic analyses. (B) Numbers of CFUs recovered from homogenates of the proximal (P), medial (M) and distal (D) segments. Bars represent the geometric mean. *, p<0.05, and **, p<0.01, based on ANOVA with Tukey's multiple comparison test. (CEG) Confocal micrographs showing VcRed and VcGreen distribution in the proximal (C), medial (E) and distal (G) segments. Tissue sections were counterstained with phalloidin (blue) to visualize the surface of the epithelium. White arrowheads mark clonal microcolonies. Scale bar = 100 µm. (DFH) The distance separating microcolonies from the base of the villi in the proximal (D), medial (F) and distal (H) SI segments was measured using confocal microscopy in tissue cross sections from mice co-inoculated with VcRed and VcGreen. Data represent the mean ±SD from three mice. The number (n) of microcolonies analyzed is indicated to the left of each panel. Black triangles indicate the average length of villi in each region. (I) Average length of intestinal villi (±SD) in the proximal (P), medial (M) and distal (D) segments. ***, p<0.001, based on ANOVA with Bonferroni's multiple comparison test.

For localization studies, equal mixtures of VcRed and VcGreen were inoculated into suckling mice. In most cases, infection was allowed to proceed for ∼24 hr, as this yields maximal colonization; however, some experiments were terminated at 8 or 16 hr, to explore earlier stages of the infection process. At each end point, the small intestines were divided into three equal parts, and total bacterial load and distribution were monitored by plating intestinal homogenates and by confocal microscopy respectively. After only 8 hr, bacteria were difficult to visualize, particularly within the proximal SI, although analyses of cfu confirmed that they were present throughout the intestine (Figure S2A). Microcolonies were not yet evident 8 hr PI (Figure S2B), and we suspect that the majority of *V. cholerae* were not yet attached to intestinal tissue this early during infection. Even at 16 hr PI, only a few small microcolonies were evident (Figure S2B); for this reason, we focused our localization analyses on the 24 PI time point. Consistent with previous analyses of cfu in both infant mice and infant rabbits [22], [24], at 24 hr post-infection (PI) *V. cholerae* were most abundant within the medial and distal thirds of the intestine, and ∼20–100-fold less abundant within the proximal third of the SI (Figure 1B). However, confocal microscopy images revealed striking and previously unrecognized features of *V. cholerae* intestinal colonization. First, we observed that *V. cholerae* microcolonies on the intestinal epithelium are nearly always uniformly red or green (Figure 1CEG and Figures S2, S3, S4), strongly suggesting that the cells in microcolonies are clonal, i.e., that microcolonies arise from a single attached bacterium and do not trap or recruit unattached bacteria as they expand.

We also detected notable differences between the distribution of microcolonies along the intestinal villi in the proximal vs the medial and distal SI segments. Unexpectedly, in the proximal SI, *V. cholerae* microcolonies were almost exclusively (>90%) located at the base of the villi, within the forming crypts (Figs. 1CD), whose development is initiated during the first postnatal week [25]. In contrast, microcolonies in the medial and distal SI, which were more numerous, were predominantly detected in the bottom halves of the ∼300 µm long villi, but only ∼30% were located at the base of the villi (Figure 1C–I). The predilection for microcolony formation at the bases of villi was not anticipated, since crypts are known to produce antimicrobial products, such as cryptidins [26]. However, such crypt-protecting defenses may not be present in the 5 day old mice used here. Notably, a majority of colonies observed on the sides of the villi appeared to occupy crevices within the intestinal epithelium, although a precise frequency was not determined (Figure 1EG, white arrowheads). Preferential localization of *V. cholerae* at the bases of villi and in crevices likely shelters the organism from peristaltic forces that would propel the pathogen towards the distal intestine.

**Figure 2:**
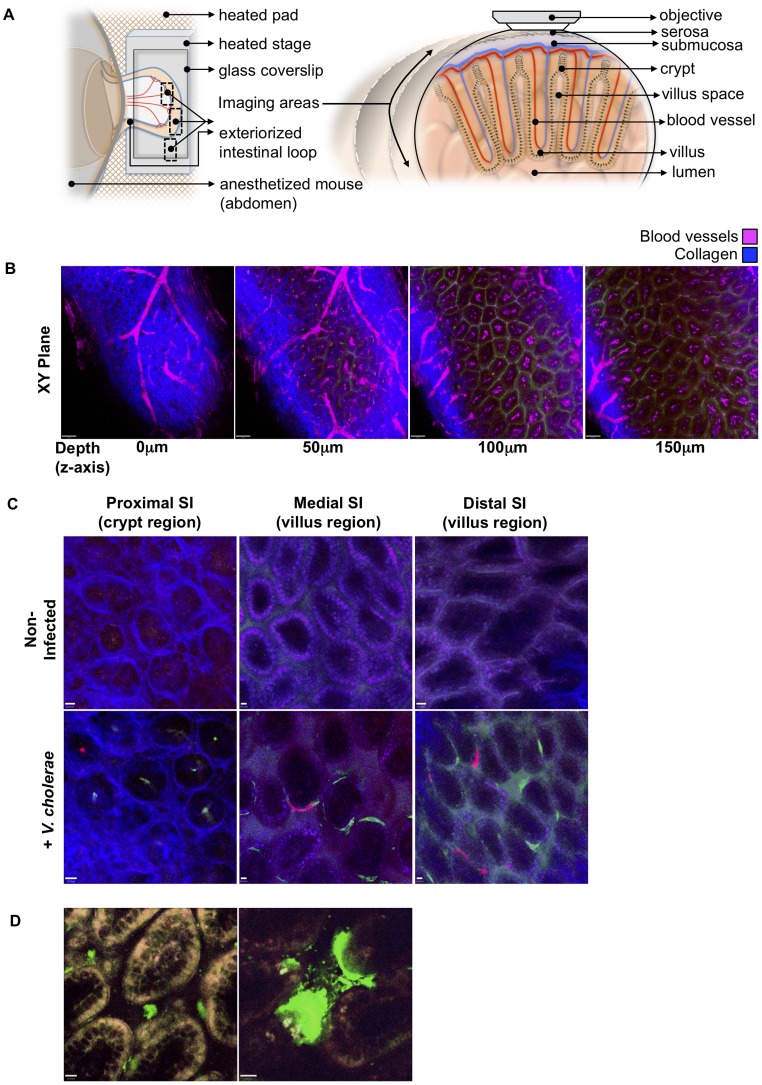
Detection of fluorescently labeled *V. cholerae* in the intact infant mouse small intestine by intravital two-photon microscopy. (a) Schematic representation (left) of the surgical intravital imaging approach to visualize intestinal tissue by two-photo microscopy in live anesthetized mice following orogastric inoculation with *V. cholerae*. Transverse view of exteriorized intestinal loop (right), depicting objective focal path from the serosal side through the various tissue layers towards the intestinal villus. The resulting focal plane is a tangential section along the intestine as depicted in (b–d). (b) Sequential two-photon micrographs (XY plane) taken from a 3-dimensional Z-stack of the SI, revealing penetration depths for two-photon excitation of up to 150 µm below the serosa layer. Depths (Z axis) 0 to 50 µm below the serosa include crypt regions, and >100 µm encompass the villi region. Blue denotes collagen fibers, Magenta denotes blood vessels. Scale bar 50 µm. (c) Differential localization of *V. cholerae* within the crypt or villus regions in the proximal, medial, and distal segments of the SI. Top row panel displays representative intravital two-photon micrographs of the crypt region for the proximal SI segment, and of the villus region for the medial and distal SI segments, of non-infected mice. Bottom row panel displays representative intravital two-photon micrographs of the crypt region and villus regions for the proximal, medial, and distal regions of the SI following orogastric inoculation with VcGreen and VcRed, respectively. Blue denotes collagen fibers, Green denotes colonies of VcGreen, Red denotes colonies of VcRed. Scale bar 10 µm. (d) Visualization of VcGreen in explanted distal SI segments. Representative two-photon micrographs taken from time-lapsed movies (see Supplemental Video S1, Video S2) show single VcGreen cells in the intravillus space and discrete microcolonies of VcGreen associated with the villus epithelium. White/Yellow denotes autofluorescence, Green denotes VcGreen. Scale bar 10 µm.

Our observations that microcolonies are largely clonal and have distinct localization in the proximal SI vs. the medial and distal SI were confirmed using intravital two-photon microscopy. In contrast to the confocal microscopy-based imaging, which requires dissection and processing (i.e. fixation and washing) of SI segments, intravital microscopy is performed using intact tissue, and thus is less likely to perturb pathogen localization. For our experiments, segments of small intestines of anesthetized infected or mock-infected suckling mice were exteriorized from the peritoneal cavity and placed on a microscope stage, and intestinal contents were visualized from the exterior of the tissue (Figure 2A). With this protocol, we could image microcolonies and tissue structure as far as ∼150 µm from the intestinal wall (Figure 2BC), which permits analysis from the serosa through the basal half of the villi, but not into the intestinal lumen. Twenty four hr after inoculation of infant mice with VcGreen and VcRed, small monoclonal colonies of either VcRed or VcGreen were detected only in crypts in the proximal small intestine; larger colonies were observed at the bases and along the bottom third of villi in the medial and distal segments of the small intestine (Figure 2C). These observations closely mirror the findings obtained with confocal microscopy, and thus provide support for the idea that *V. cholerae* microcolonies have distinct distributions in different segments of the intestine.

We also imaged explanted SI segments from VcGreen infected animals with two-photon microscopy. The explants (which were not opened *en face*) were mounted in a saline/lubricant gel imaging chamber that enables enhanced visualization of the intervillous space. In this setting, we were able to detect individual VcGreen cells moving through the intervillous spaces and occasionally contacting the large attached microcolonies that were particularly prominent in these images (Figure 2D and Videos S1, S2). Although the movement of VcGreen cells may reflect external convective forces rather than intrinsic bacterial motility, these images suggest that it may be possible to analyze the interactions of single tagged *V. cholerae* cells with each other and with the epithelium in future studies.

**Figure 3:**
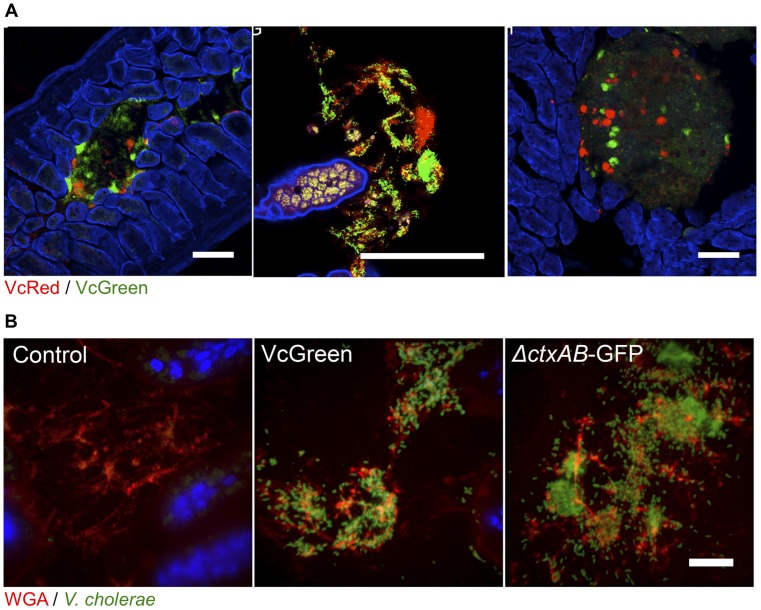
*V. cholerae* aggregates are present in the lumen of the lower SI, often associated with mucus. (A) Each panel shows lumenal aggregates of *V. cholerae* (VcRed and VcGreen) from the medial or distal SI. Colonies in the right panel reside on the surface of digesta. Scale bar = 100 µm. (B) Confocal microscopy images of tissue sections from the lower SI of infant mice inoculated with LB (control), VcGreen or the GFP-labeled cholera toxin–deficient mutant *ΔctxAB*-GFP. Sections were counterstained with wheat germ agglutinin (red) to visualize mucus and DAPI (blue) to stain nuclei. Scale bar = 25 µm.

Although luminal (unattached) bacteria cannot be monitored using two-photon microscopy, luminal *V. cholerae* were observed in the medial and distal segments of the SI using confocal microscopy. These bacteria were often present as large clonal (all green or all red) aggregates, but mixed populations of VcRed and VcGreen were observed as well (Figure 3A). In the distal SI, clonal microcolonies were detected on the surface of digesta, suggesting that *V. cholerae* may adhere to and grow upon luminal contents (Figure 3A).

We also visualized tissue sections from mice inoculated with a single marked strain (either VcGreen or the cholera toxin-deficient mutant, ΔctxAB-GFP) that were stained with wheat-germ agglutinin (WGA), a lectin that binds to terminal N-acetyl-D-glucosamine and sialic acid residues on sugar chains [27]. WGA allows visualization of the highly glycosylated mucins in the glycocalyx that lines the epithelial brush border surface and that constitute intestinal mucus. Luminal *V. cholerae* colonies were often embedded in a WGA-rich matrix (Figure 3B). As was previously seen in infant rabbits infected with VcGreen, these clumps are reminiscent of the *V. cholerae*/mucus aggregates found in the ‘rice-water’ stool of cholera patients. Interestingly, in the rabbit model, luminal mucus accumulates in response to cholera toxin, which induces release of mucins from intestinal goblet cells [24]; however, in infant mice, the luminal WGA-reactive material was also present in uninfected control mice. Furthermore, and, in contrast to observations in *V. cholerae*-infected infant rabbits, no obvious difference between the amounts of luminal WGA-reactive material was observed in mice infected with VcGreen vs its colonization proficient but toxin-deficient Δ*ctxAB* counterpart. (Figure 3B). Thus, in infant mice, the WGA-stained matrix in which *V. cholerae* is embedded does not appear to be derived from mucins released by goblet cells in response to cholera toxin.

#### Mucin abundance decreases along the length of the SI

**Figure 4:**
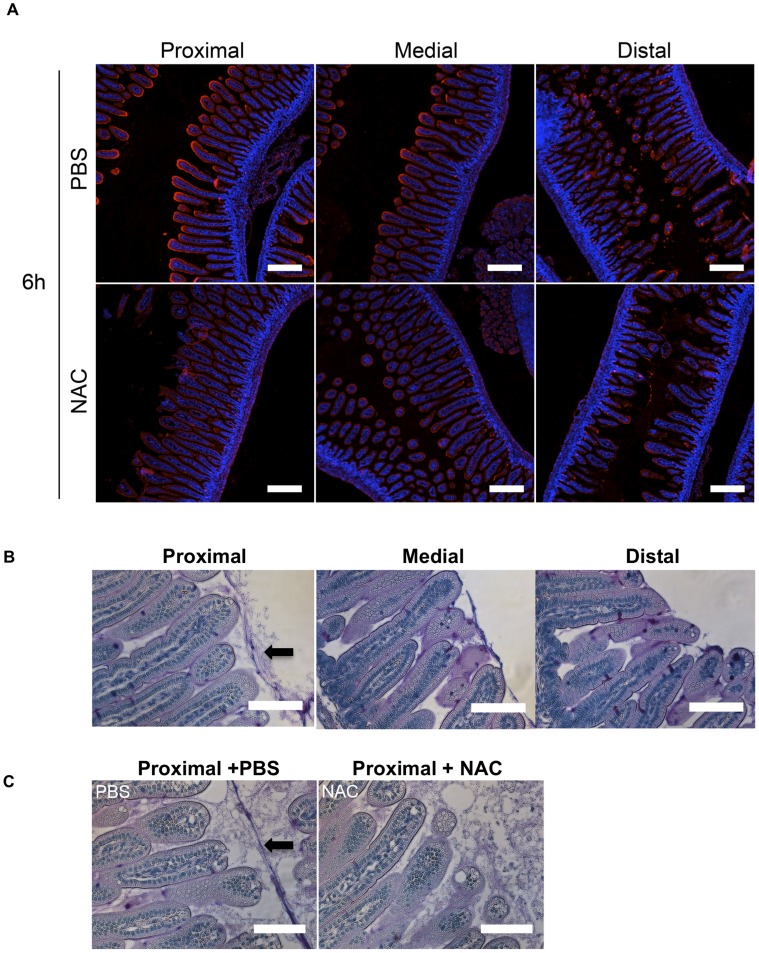
The intensity of WGA staining of the intestinal epithelium decreases along the length of the SI and can be reduced by N-acetyl-L-cysteine (NAC) treatment. (A) Confocal micrographs of longitudinal sections of the proximal, medial and distal SI from infant mice treated with PBS or NAC after 6 h. The sections were stained with WGA (red) and DAPI (blue). Scale bar = 200 µm. (B,C) PAS stained sections of Carnoy's fixed proximal, medial and distal SI segments from untreated infant mice (B) and of the proximal SI from infant mice treated with PBS or NAC after 6 h (C). Scale bar = 100 µm. Arrow points to mucus layer.

WGA staining also revealed extensive reactivity of the epithelial surface in the proximal and middle SI, but not in the distal SI (Figure 4A, PBS). This result suggests that either the cell-associated glycocalyx or the unattached mucus layer decreases in abundance along the length of the SI; however, since formaldehyde fixation is thought to shrink the mucus layer, so that it collapses onto the underlying tissue and loses its distinct identity, it was not possible to distinguish between these possibilities. Therefore, we also examined SI samples fixed in Carnoy's solution (which yields enhanced preservation of the mucus layer [28]) and stained with Periodic acid schiff (PAS), which detects polysaccharides, such as carbohydrate-rich mucins. In tissue treated with Carnoy's/PAS, a mucus layer covering the intestinal villi was generally detectable in all 3 parts of the SI, but tended to be thicker and more continuous in the proximal part of the SI compared to the more distal parts (Figure 4B). These results raise the possibility that differential abundance of mucins along the length of the intestinal tract may be a factor in *V. cholerae's* preferential localization in the distal portion of the SI.

### Differential requirement for flagellar-based motility for colonization along the SI

**Figure 5:**
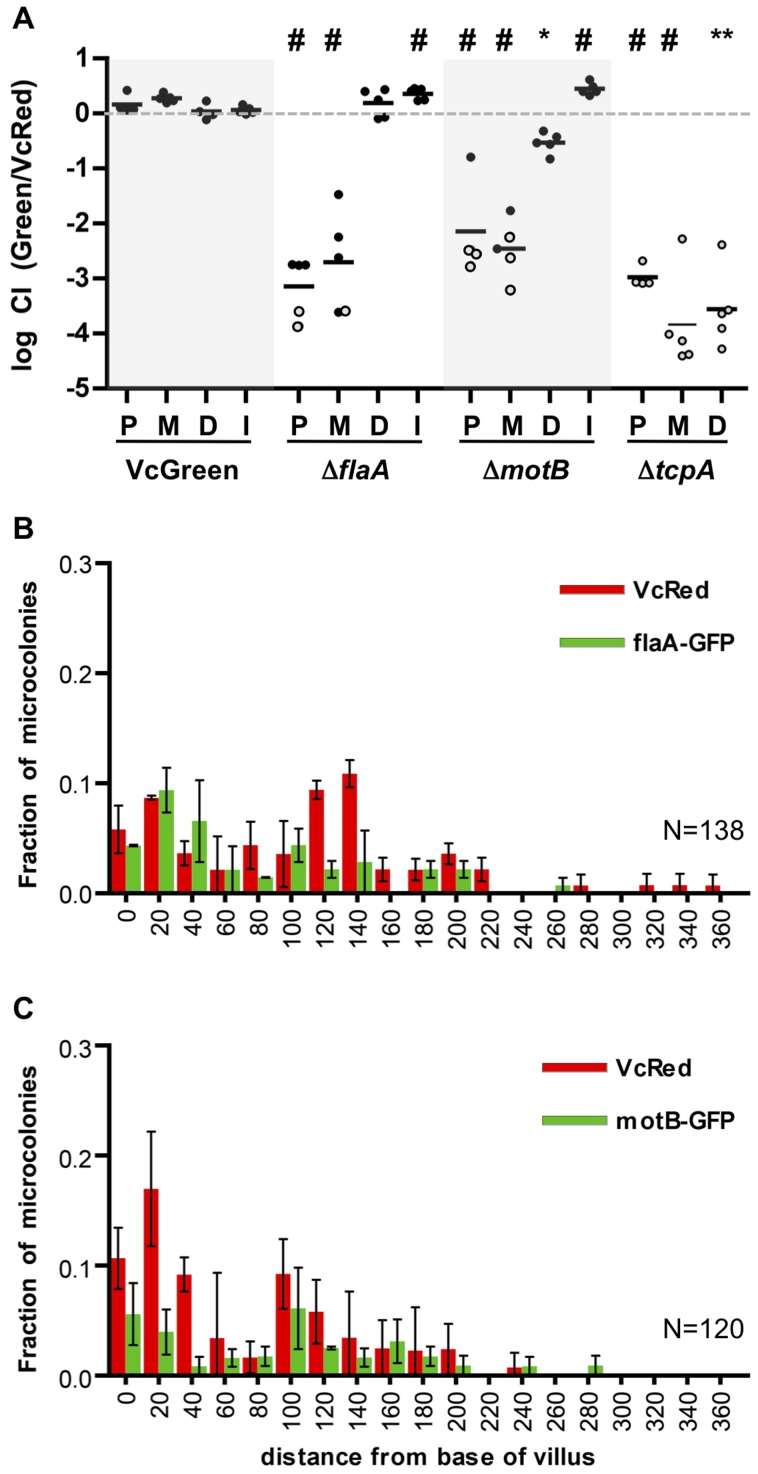
Differential requirements for flagellar-based motility along the SI. (A) Competitive indices (CI) from competition assays using GFP-labeled C6706, *ΔflaA*, *ΔmotB* or *ΔtcpA* and VcRed in the proximal (P), medial (M) and distal (D) SI segments and in vitro (I). Bars represent the geometric mean. *P<0.05, **P<0.01, #P<0.001, based on ANOVA followed by Bonferroni's multiple comparison post test, comparing the data with the corresponding VcGreen/VcRed samples. Open symbols mark the limit of detection for animals from which no mutants were recovered. (B,C) Distribution of *ΔflaA*-GFP (B) and *ΔmotB*-GFP (C) vs VcRed microcolonies along the axis of intestinal villi in the distal SI segment. The distance separating microcolonies from the base of the villi was measured by confocal microscopy in tissue cross sections from three mice. Data represent the mean ±SD. The number (n) of microcolonies analyzed is indicated in the bottom right of each panel.

To begin to understand the determinants of *V. cholerae* localization within the SI, we investigated the impact of disrupting bacterial or host processes that might contribute to bacterial localization, including bacterial motility and chemotaxis and the host mucus layer. In previous analyses, enumeration of *V. cholerae* in homogenates of the entire suckling mouse SI revealed that motility-deficient *V. cholerae* strains have a reduced capacity to colonize [12], [13], [29], [30], perhaps because flagellar-based motility enables the pathogen to reach particular intraintestinal sites; however, with the exception of one early study using undefined non-motile *V. cholerae* mutants [31], the impact of flagellar-based motility upon bacterial localization within the intestine has not been reported. Therefore, we carried out in vivo competition assays using VcRed and a GFP-marked *ΔflaA V. cholerae* mutant, which lacks the major flagellin subunit and does not produce a flagellum [32], [33]. As found in previous studies, the non-flagellated strain displayed a colonization defect, but notably, the effect of the mutation was not uniform across the small intestine. Instead, colonization was reduced (relative to the wt strain) by ∼1000-fold and 500-fold in the proximal and medial SI segments, but unimpaired in the distal segment (Figure 5A). To exclude the possibility that the flagellum might promote colonization via mechanisms independent of motility, such as enhancing adhesion, a GFP-marked non-motile but flagellated strain lacking the MotB component of the flagellum motor (*ΔmotB*-GFP) [32] was also tested in in vivo competition assays. Similar to the *ΔflaA* mutant, the *ΔmotB* mutant was markedly defective at colonizing the proximal and medial segments of the small intestine, but it also exhibited a modest colonization defect (5-fold) in the distal SI (Figure 5A). The similar phenotypes of the *ΔflaA* and *ΔmotB* mutants are consistent with the idea that the colonization defect of the *ΔflaA* mutant is due to its motility deficiency. To our knowledge, flagellar motility is the first *V. cholerae* attribute shown to be required for colonization of only a subset of intestinal sites. Typically, colonization factors are required throughout the intestine, as we observed for a TCP-deficient mutant (*ΔtcpA*), which exhibits highly compromised colonization in all SI segments (Figure 5A). Our data suggests that flagellar-based motility is critical for *V. cholerae's* ability to reach and/or be maintained in the proximal ∼2/3 of the SI, but that it is relatively unimportant for infection of the distal third of the SI.

The distribution of the non-motile strains was also assessed using confocal microscopy. Consistent with the findings from the plating assays discussed above, neither GFP-marked *ΔflaA* or *ΔmotB V. cholerae* were visible in the proximal SI (Figure S5), and colonies were rare in the middle SI as well. Surprisingly, the absence of flagellar-based motility did not dramatically alter the distribution of *V. cholerae* in the distal SI; *ΔflaA* and *ΔmotB* colonies were detected both at the base of villi and at lateral positions (Figure 5BC), suggesting that *V. cholerae* cells do not depend on flagellar-based motility to penetrate into intervillous spaces in the distal SI, as has previously been proposed [16]. Since functional flagella are also required for chemotaxis, these data also suggest that *V. cholerae* does not depend upon chemotaxis to penetrate into the intervillous spaces within the distal SI of infant mice.

### Chemotaxis is not required for microcolony localization along the villous axis

**Figure 6:**
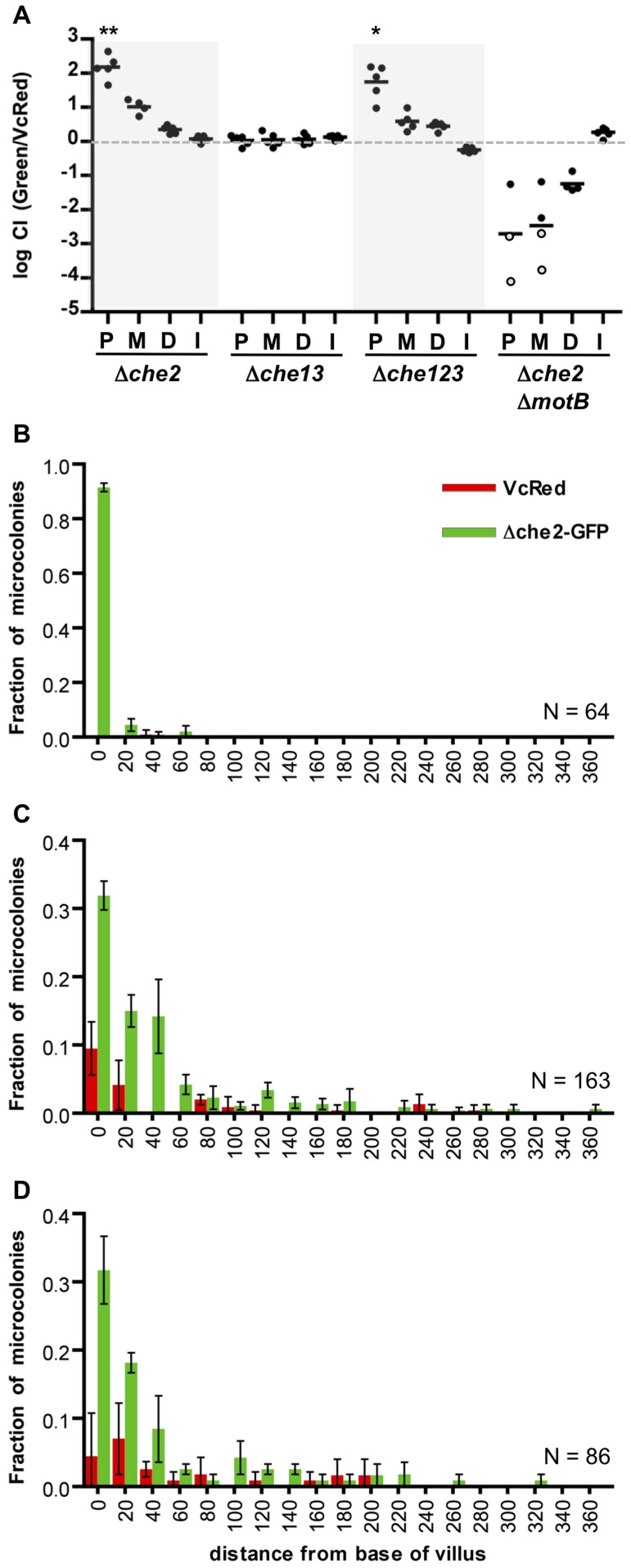
Chemotaxis is not required from the fine localization of *V. cholerae* microcolonies along the villous axis. (A) Competition assays between GFP-labeled chemotaxis cluster deletion mutants *Δche2* (cluster 2), *Δche13* (clusters 1 and 3) or *Δche123* (all 3 clusters) and VcRed in the proximal (P), medial (M) and distal (D) segments and in vitro (I). Bars represent geometric means. *P<0.05, **P<0.001, based on ANOVA followed by Bonferroni's multiple comparison post test, comparing the data with the corresponding VcGreen/VcRed samples. (B–D) Distribution of *Δche2*-GFP and VcRed microcolonies along the axes of intestinal villi in the proximal (B), medial (C), and distal (D) intestine. The distance separating microcolonies from the base of the villi was measured by confocal microscopy in tissue cross sections from three mice co-inoculated with GFP-labeled *Δche2* and VcRed. Data represent the mean ±SD. The number (n) of microcolonies analyzed is indicated in the bottom right of each panel.

We performed similar analyses of the colonization and intestinal distribution of *V. cholerae* lacking various chemotaxis genes. *V. cholerae* contains 3 gene clusters that encode homologues of chemotactic proteins, one of which (cluster 2) is known to be required for chemotaxis in vitro [14] [13], [15]. Inactivation of particular cluster 2 genes can lead to enhanced colonization of the infant mouse intestine, especially but not exclusively in the proximal SI [13], [17]. Roles for chemotaxis clusters 1 and 3 have not yet been defined. Consistent with previous observations of hypercolonization by a mutant lacking *cheY3* or *cheA2* (components of cluster 2) [13], [17], we found that a *V. cholerae* strain harboring a deletion of the entire set of cluster 2 genes (*Δche2*) out-competed the wt strain ∼100× and ∼10× in the proximal and medial SI segments respectively (Figure 6A). In contrast, colonization by a mutant lacking the other 2 clusters (*Δche13*) did not differ from that of the wt strain (Figure 6A). A triple mutant harboring deletions of all 3 putative chemotaxis clusters (*Δche123*) exhibited hypercolonization indistinguishable from the *Δche2* mutant (Figure 6A), providing further evidence that the products of clusters 1 and 3 do not contribute to colonization, even in a secondary role. Notably, the hyper-colonization phenotype of the *Δche2* mutant was disrupted by inactivation of *motB*, suggesting that bacterial motility is required for hypercolonization, even though the motility of the *Δche2* mutant is undirected (Figure S6). The *Δche2ΔmotB* mutant exhibited a colonization defect similar to the *ΔmotB* strain in all parts of the SI (Figure 6A).

To further assess the importance of chemotaxis in promoting *V. cholerae*'s capacity to navigate into and through the intervillous spaces, we monitored the distribution of *Δche2*-GFP microcolonies along the villous axis in the different SI segments. Notably, the fine localization of *Δche2*-GFP strain was very similar to that of wt *V. cholerae* in all intestinal segments, despite the markedly increased number of cfu in some segments. Like VcGreen and VcRed, in the proximal SI, nearly all *Δche2*-GFP microcolonies were found at the bases of villi, though they were found with much higher numbers than the chemotaxis-proficient bacteria (Figure 6B). No notable differences in the sizes of *Δche2* and WT microcolonies were observed, suggesting that the hypercolonization of *Δche2* is likely explained by the elevated number of crypts occupied by this mutant (although this remains a small fraction of crypts overall). In the medial and distal SI segments, *Δche2*-GFP was found at the base of villi and along the lower third of villus surfaces, as also was observed for VcRed (Figure 6B, note in the medial segment that *Δche2*-GFP significantly outcompetes VcRed). Thus, our results indicate that *V. cholerae*'s only known functional chemotaxis cluster does not guide its fine localization in the small intestine, and counter the long-standing hypothesis that *V. cholerae* chemotaxis directs the organism toward the crypts [16], [34]. Additionally, our results suggest that hypercolonization by the *Δche2*-GFP strain does not reflect occupancy of a novel niche within the proximal SI; instead, in the absence of chemotaxis, *V. cholerae* simply establishes microcolonies within a higher percentage of proximal SI crypts than are occupied by wt bacteria.

### Mucus contributes to the colonization resistance of the proximal SI

**Figure 7:**
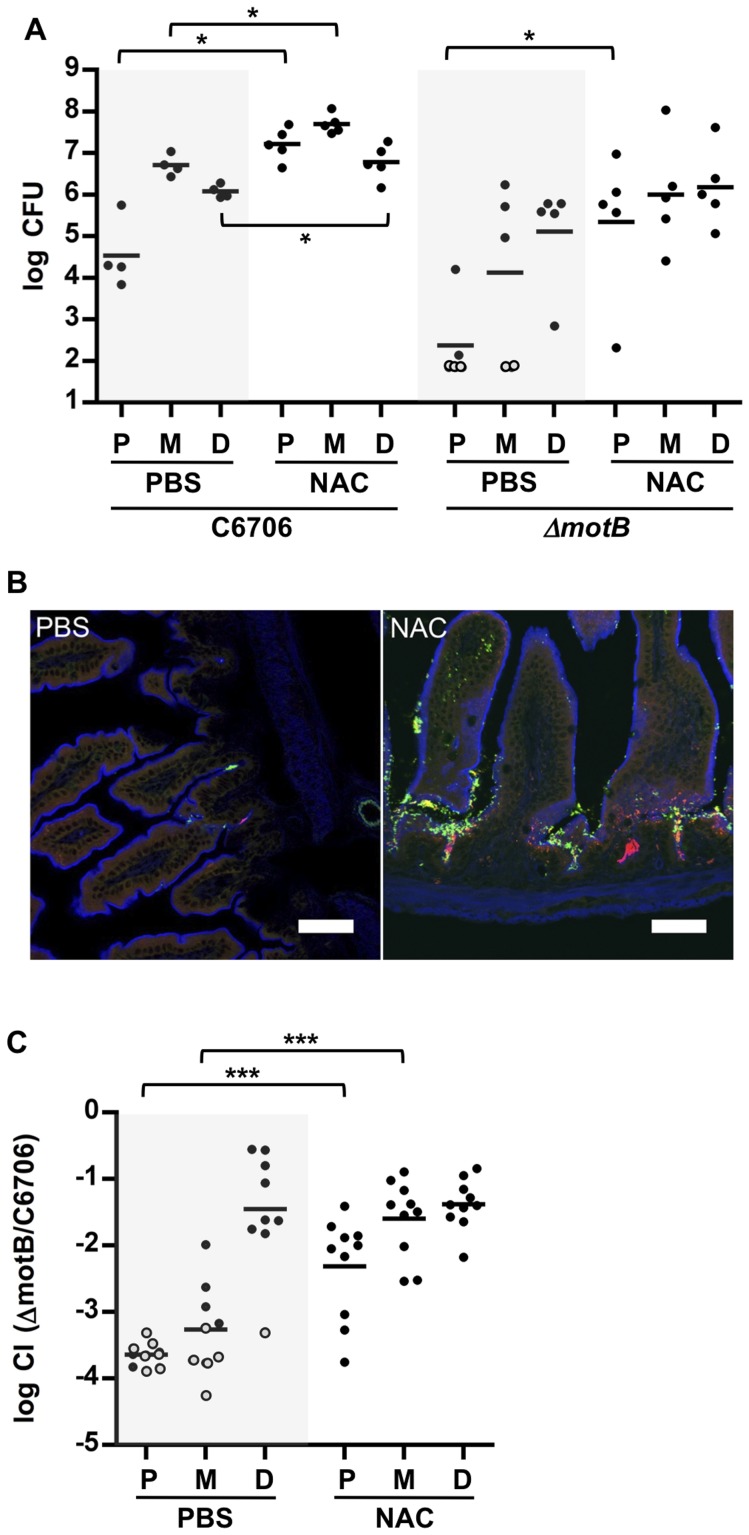
NAC pretreatment promotes *V. cholerae* intestinal colonization and reduces the requirement for motility. (A) Numbers of CFUs recovered from homogenates of the proximal (P), medial (M) and distal (D) segments of infant mice treated with PBS (black) or NAC (red) 30 minutes prior to inoculation with C6706 or *ΔmotB*. Bars represent the geometric mean. *P<0.05; Mann-Whitney test. (B) Confocal micrographs of tissue sections of the proximal SI from infant mice treated with PBS or NAC, 30 minutes prior to co-inoculation with VcGreen and VcRed. Scale bar = 50 µm. (C) Competition assays between GFP-labeled *ΔmotB* and VcRed in the P, M and D segments from mice treated with PBS or NAC 6 h prior to bacterial inoculation. Bars represent the geometric mean. ***P<0.001; Mann-Whitney test.

To investigate whether the more abundant mucus layer in the proximal SI contributes to the relative resistance of this region to *V. cholerae* colonization, we treated mice with the mucolytic agent N-acetyl-L-cysteine (NAC), which is thought to disrupt the disulfide bonds between mucins [35]. Six hours after NAC was introduced by gavage into infant mice, there was marked reduction in WGA staining on the surface of intestinal villi (Figure 4A, NAC); in addition, this treatment appeared to partially disrupt and disorganize the mucus layer detected with PAS staining of Carnoy's fixed samples (Figure 4C). The effects of NAC were reversible, and by 24 hr after NAC treatment, staining was restored to pre-treatment intensity (Figure S7). Notably, pre-treatment of mice with NAC 30 minutes before *V. cholerae* inoculation increased colonization of all SI segments, but particularly the proximal SI. Nearly 150-fold more *V. cholerae* CFU were recovered from the proximal SI of NAC treated mice than from control (PBS-treated) animals (Figure 7A). Furthermore, confocal imaging revealed *V. cholerae* along the villi as well as at the base of villi in the proximal SI of NAC treated mice, rather than solely within crypts (Figure 7B). Increased colonization was also detected for the medial and distal SI (∼10× and ∼6×, respectively; Figure 7A). Overall, NAC treatment largely abolished differential colonization of SI regions, suggesting that mucus is a key factor in countering intestinal colonization by *V. cholerae*. NAC is also known to function as an antioxidant, and is possible that NAC also promotes bacterial growth by reducing the level of reactive oxygen species (ROS) in the intestinal lumen; however, NAC appears to be most potent against intracellular ROS [36].

Additional experiments with NAC treated mice suggest that the inability of the motility deficient *ΔmotB V. cholerae* mutant to penetrate the mucus barrier accounts for a significant portion of this strain's colonization deficiency. In both single infection and competitive infection experiments, the capacity of the *ΔmotB* strain to colonize the intestines of untreated mice is lower than that of the wt by ∼1 to several orders of magnitude, with the largest deficiency seen in the proximal intestine (Figure 4 and Figure 7AC). However, NAC treatment promoted colonization by the *ΔmotB* mutant, particularly in the proximal intestine, resulting in much less marked attenuation compared to the wt strain (Figure 7AC). Thus, although directed (i.e., chemotaxis-based) movement is not required for establishment of an infection, the bacterial ability to propel itself through, or escape from, mucus, seems to play a significant role, at least in the proximal intestine, where the mucus layer appears to be most prominent.

## Discussion

Using confocal and two-photon microscopy to detect fluorescent *V. cholerae* in the suckling mouse intestine, we have obtained new insights regarding where and how this pathogen grows in the host, as well as the bacterial and host processes that modulate colonization. Direct visualization of the pathogen within the intestine suggests that the majority of *V. cholerae* microcolonies observed on the intestinal epithelium arise from single attached cells; expansion of such colonies likely accounts for a significant proportion of *V. cholerae* proliferation within the host environment. Visualization of the pathogen also uncovered unexpected and striking differences between the fine localization of *V. cholerae* microcolonies within distinct regions of the SI. Notably, microcolonies were found almost exclusively in the developing crypts in the proximal intestine but at the bases and along the bottom third of villi in the distal 2/3 of the SI. The predilection of *V. cholerae* to occupy the crypts, the lower parts of villi and crevices within villi, likely provides a means for the pathogen to avoid the propulsive force of intestinal motility, which directs ingested material and secreted fluid and mucus toward the distal intestine. Residency in crypts may particularly protect bacteria against being shed from the epithelial surface. Host mucus seems to be a key factor that limits *V. cholerae* intestinal colonization, particularly in the proximal SI where there appears to be a thicker mucus layer.

Surprisingly, *V. cholerae* motility proved to have a regiospecific influence on intestinal colonization. Nonmotile mutants failed to colonize the proximal SI but were not compromised in their capacity to colonize the distal SI, where their distribution was similar to that of wt *V. cholerae*. It is possible that motility is required to penetrate the mucus layer, as originally proposed by Guentzel et al decades ago [31], since NAC treatment partially alleviated the colonization defect of the motility-deficient *motB V. cholerae* mutant. However, since NAC treatment also augments colonization by wt bacteria, it is likely that mucus imposes a barrier to colonization by wt *V. cholerae* as well. The relative lack of mucus in the distal SI may at least in part explain why the motility-deficient strains retained the capacity to colonize this part of the SI, and may also contribute to the preferential colonization of this region by wt *V. cholerae*. However, it is important to note that despite the impact of motility on the gross distribution of *V. cholerae* in the SI, motility is dispensable for the pathogen's proper fine localization in the distal SI. These results raise the possibility that flagellar motility enables *V. cholerae* dissemination throughout the lumen of the small intestine, but that additional (non-flagellum based) processes control its penetration into the intervillous space. Such processes could include peristalsis, mucus structure/organization and the distribution of (currently unknown) host targets of *V. cholerae* adhesions. In addition, *V. cholerae* has been reported to possess flagellum-independent motility on surfaces [37], and it has been proposed that flagellum-independent motility may aide *V. cholerae* migration through intestinal mucus [38].

Our findings counters the long-standing hypothesis, developed more than 30 years ago in pioneering studies by Freter, that chemotaxis facilitates *V. cholerae* penetration deeper into the intestinal mucosa and intervillous space, and that such penetration results in bacterial killing, due to the presence of unknown antimicrobial factors [16]. We demonstrate that although chemotaxis-deficient *V. cholerae* has an enhanced capacity to colonize the upper SI, its fine localization in both the upper and lower SI is equivalent to that of wt *V. cholerae*. The abundant nonchemotactic *V. cholerae* detected in the upper SI reside entirely within the crypts, clearly demonstrating that *V. cholerae* does not need chemotaxis to penetrate into the deepest zones of this tissue. Thus, like motility, chemotaxis appears to play a more prominent role in the overall distribution of *V. cholerae* within the intestine than in its fine localization within intestinal segments. As noted by Butler and Camilli [17], the tendency of non-chemotactic mutants to be biased towards straight swimming may help them to enter new intestinal sites and may contribute to their colonization phenotype. Indeed, such altered swimming could potentially have more impact than an inability to respond to either positive or negative chemotactic stimuli. Consistent with this possibility, we observed that the hypercolonization associated with the Δ*che2* mutation is dependent upon flagellar motility; a Δ*che2 motB* mutant did not exhibit hypercolonization.

Both the distribution of host glycans and the effects of NAC treatment support the idea that host mucins restrict *V. cholerae* localization along the SI as well as along the villous axis. NAC treatment rendered the proximal SI much more permissive to *V. cholerae* colonization; it enabled the pathogen to occupy new sites along the villous axis in this intestinal region. Intestinal mucins are thought to constitute a key host defense against a variety of enteric pathogens [21], and many commensals and pathogens, including *V. cholerae*, produce enzymes (e.g the ToxR-regulated TagA mucinase [39]) that cleave sugars from or the peptide backbone of mucins. Although host mucus likely serves as a physical barrier between *V. cholerae* and intestinal tissue that limits infection, it is also likely to be an important source of energy for *V. cholerae* and other enteric pathogens that can digest its carbohydrate components. Previous studies have already revealed that a *V. cholerae* sialidase promotes robust *V. cholerae* colonization [40], and we observed that *V. cholerae* in the intestinal lumen is often associated with intestinal mucus. It should be possible to use fluorescence microscopy-based approaches along with genetically engineered mice (e.g., mutants unable to glycosylate the principal secreted mucin, MUC-2) and wt and mutant *V. cholerae* to further characterize the interplay between host mucins and this pathogen.

Finally, our observations of SI segments with intravital two-photon microscopy, a technique that does not perturb host tissues, corroborated our findings with confocal microscopy, which requires tissue processing. Like the confocal images, the two-photon images revealed that *V. cholerae* microcolonies are primarily monoclonal and showed differences between the fine localization of *V. cholerae* along the villous axis in different parts of the SI. To our knowledge, these observations represent the first application of intravital microscopy to imaging an orogastrically inoculated enteric pathogen in an intact intestine. Previous intravital imaging of enteric pathogens have relied on surgical exposure of the intestinal lumen and have primarily focused on interactions of pathogens with dendritic cells/macrophages (e.g. [41] [42]). Our findings suggest that it should be possible to use intravital microscopy to monitor host-pathogen and potentially pathogen-pathogen and pathogen-commensal interactions that occur on intestinal epithelial surfaces in real time.

## Materials and Methods

### Bacterial strains

All *V. cholerae* strains used in this study are streptomycin-resistant derivatives of C6706, a 1991 El Tor O1 Peruvian clinical isolate. The *ΔflaA*, *ΔmotB*, *ΔtcpA*, and *ΔctxAB* strains have been described previously [24], [32]. The chemotaxis operon deletion strains *Δche2* (strain SR28, *Δvc2059-vc2065*), *Δche13* (strain SR31, *Δvc1394-1406* (che1), *Δvca1088-vca1096* (che2)) and *Δche123* (strain SR33, *Δvc1394-1406 (che1), Δvc2059-vc2065 (che2), Δvca1088-vca109*6 (che3)) were created by allelic exchange as described in [43], [44]. GFP-labeled strains, which constitutively express GFPmut3 under the control of the lac promoter, were generated by introducing the suicide vector pJZ111 (a kind gift of Dr. Jun Zhu) into the *lacZ* locus as described [24]. A derivative of pJZ111 (pYM50) was generated by inserting a *V. cholerae* codon-optimized version of the tdTomato gene (Genscript) in place of the GFPmut3 gene. This plasmid was used to generate the strain VcRed, which constitutively expresses tdTomato.

### Infection assays

5-day old CD-1 mice were intragastrically inoculated as described [22]. For in vivo competition assays, 1∶1 mixtures of a GFP-labeled strain and VcRed were inoculated into each mouse (∼2×10^5^ cells/mouse). After 24 h, unless otherwise noted, animals were euthanized and their small intestines removed and divided into three parts of equal length (proximal, medial and distal, ∼3.5 cm each); the central 1 cm segment of each part was removed, homogenized in LB and plated. For in vitro competition assays, 5 mL of LB containing streptomycin (200 µg/mL) were inoculated with 10 µL of the in vivo inoculum and grown at 30°C for 24 h. Serial dilutions were then plated. The number of CFUs of the GFP-labeled strain were determined by scanning the plates using a fluorescent image analyzer (Fujifilm FLA-5100). The ratio between GFP-labeled and VcRed CFUs was calculated and normalized by the ratio in the inoculum to determine the competitive index (CI). For single infection assays, ∼2.10^5^ cells were inoculated into each mouse and after 24 h, the SI segments were prepared and processed as described above.

### Statistical analyses

Statistical analyses were performing with Prism (GraphPad).

### Ethics statement

This study was performed in strict accordance with the recommendations in the Guide for the Care and Use of Laboratory Animals of the National Institutes of Health. All animal protocols were reviewed and approved by the Harvard Medical Area Standing Committee on Animals (protocol #04316).

### Analysis of *V. cholerae* intestinal localization using confocal microscopy

Tissue from a subset of mice used in infection studies was analyzed via confocal microscopy (n = 3 per assay). Mice were inoculated with VcRed and/or a GFP-labeled strain as described above. Tissue samples from the proximal, medial, and distal intestine were fixed in PBS with 2% paraformaldehyde for two hours at room temperature (RT), placed in PBS with 30% sucrose for two hours at RT, mounted in tissue freezing medium (EMS), snap-frozen in dry ice-cold 2-metylbutane and sectioned (10 µm). Initially, bacteria labeled with GFP were visualized via direct detection of the fluorescent protein; however, however, we found that these signals were less stable than those obtained via immunodetection of GFP, and so most images presented here were generated via immunostaining. No difference was detected between bacterial localization observed with the two approaches. For staining, frozen sections were washed in PBS for 5–15 minutes at RT, blocked in blocking buffer (1% BSA, 5% normal donkey serum in PBS) for 1 hour at RT, stained with a primary anti-GFP antibody (Abcam, ab13970) 1/1000 in blocking buffer with 0.2% tween20 for 1 hour at RT, washed three times in PBS, stained with a FITC-coupled secondary antibody (Abcam, ab6873) 1/1000 in blocking buffer with 0.2% tween20 for 1 hour at RT, washed three times in PBS, counterstained with DAPI (1 µg/mL) and in some cases with phalloidin-alexa fluor 647 or wheat germ agglutinin (WGA)-alexa fluor 633 1/1000 (Life Technologies) for 20 min at RT and washed twice in PBS. Slides were mounted in fluorsave (calbiochem) and observed under an Olympus FluoView confocal microscope using a 20× objective or a Nikon Perfect Focus spinning disc confocal microscope. Multiple images were collected per section. Distances separating microcolonies from the base of the villi were measured using the imaging software Imaris.

### Intravital two-photon microscopy

Mice were anesthetized with ketamine, xylazine, and acepromazine and placed in a supine position on a temperature-controlled heating pad. An ∼1.2 cm vertical incision was made along the midline of the abdomen through the skin and peritoneal membrane to expose the peritoneal cavity. A 1 cm loop of small intestine (proximal, medial, or distal segment) was carefully exteriorized through the peritoneum using cotton-tipped applicators to avoid tissue damage, and lightly immobilized with tissue-adhesive glue onto a heated stage. For intravital imaging, the intestinal loop was not opened along the antimesenteric border but rather left intact for the duration of the imaging procedure. Importantly, this approach best-preserved the physiology of the small intestine, including maintaining intact blood and lymphatic flow. The intestinal loop was kept hydrated by overlaying a mixture of saline/lubricant gel, and covered by a glass coverslip. Mice were given Hoechst 33342 (Sigma; 10 mg/kg i.v.) for nuclear staining in vivo, or Qtracker-655 non-targeted quantum dots (Invitrogen; 0.2 uM i.v.) to label the vasculature in vivo. In some experiments, segments of the small intestine were occluded at either end with sutures, and then surgically removed and imaged as an explant in a heated imaging chamber containing a mixture of saline/lubricant gel and covered by a glass coverslip. Time-lapse or static imaging was performed using an Ultima Two-Photon Microscope (Prairie Technologies). Two-photon excitation and second-harmonic signals were generated using a Tsunami Ti:sapphire laser with a 10-W MilleniaXs pump laser (Spectra-Physics), and outfitted with a 20× (0.95NA Olympus) water immersion objective. Two-photon excitation wavelength was tuned to 880–950 nm for optimal fluorescence excitation of fluorescent *V. cholerae*. Emitted light and second-harmonic signals were detected through 450/50-nm, 525/50-nm, 590/50-nm, and 665/65-nm bandpass filters for four-color imaging. Image sequences were transformed into volume-rendered z-stacks with Volocity software (Improvision) or Imaris (Bitplane).

### NAC treatment

A 100 mg/mL N-acetyl-L-cysteine (NAC) solution was prepared fresh in PBS and its pH adjusted to 7.3 with NaOH. 2 mg/g of the NAC solution or an equivalent volume of PBS (mock) was administered by gavage to 5-day old CD-1 mice.

### Periodic Acid Schiff staining of intestinal mucus

Tissue samples were fixed in freshly made Carnoy's fixative (60% ethanol, 30% chloroform 10% acetic acid) for one hour at room temperature, washed in 70% ethanol and stored in 70% ethanol until further processing. Samples were embedded in paraffin, sectioned and stained with periodic acid-Schiff (PAS) at the Dana Farber/Harvard Cancer Center Rodent Histology Core.

## Supporting Information

Figure S1 **VcGreen and VcRed exhibit WT growth in vivo and in vitro.** Competition assays between VcGreen (G) or VcRed (R) vs the parental strain C6706 in the SI of infant mice (in vivo) and in vitro in LB. Bars represent the geometric mean.
(PDF)

Figure S2 **Spatial and temporal differences in CFU recovered from the small intestine.** The small intestines of infant mice co-inoculated with VcRed and VcGreen were divided into three equal parts and the central 1 cm segments of the proximal (P), medial (M) and distal (D) parts were used for plating and microscopic analyses. (A) Numbers of CFUs recovered from homogenates of each segment at 8, 16, or 24 hr PI. Mean values and SEM are plotted. (B) Confocal micrographs showing VcRed and VcGreen distribution in the proximal and medial segments at these time points. Tissue sections were counterstained with DAPI (blue) and phalloidin (gray). Scale bars = 50 µm.
(PDF)

Figure S3 **Confocal micrographs of intraintestinal VcRed and VcGreen.** Tissue from the medial small intestines of animals coinfected with VcRed and VcGreen for 24 hr was stained with DAPI (blue) and phalloidin (gray) Individual channels showing DAPI (B, G), VcGreen (C, H), VcRed (D, I), and phalloidin (E, J) are shown, as well as merged images (A, F). Scale bars, 50 µm.
(PDF)

Figure S4 **High magnification confocal micrographs of intraintestinal VcRed and VcGreen, showing individual cells.** Tissue from the small intestine of animals coinfected with VcRed and VcGreen for 24 hr was stained with DAPI (blue) and phalloidin (gray). Individual channels showing DAPI (B, G), VcGreen (C, H), VcRed (D, I), and phalloidin (E, J) are shown, as well as merged images (A, F). Scale bars, 10 µm (A–E) and 25 µm (F–J).
(PDF)

Figure S5 **Distribution of** ***ΔflaA*** **and** ***ΔmotB*** **microcolonies along the axes of intestinal villi in the proximal and distal SI segments.** The distance separating microcolonies from the base of the villi was measured by confocal microscopy in tissue cross sections from three mice co-inoculated with GFP-labeled *ΔflaA* or *ΔmotB* and VcRed. Data represent the mean ±SD. The number (n) of microcolonies analyzed is indicated in the bottom right of each panel.
(PDF)

Figure S6 **The frequency of reversals in swimming direction of the Δche2 mutant is reduced compared with the wild type. Images of swimming wild type and Δche2 mutant cells (A) and reversal frequency/sec (B).** Images of swimming cells were recorded and analyzed as previously described [41].
(PDF)

Figure S7 **The influence of NAC treatment is no longer detectable 24 h after treatment.** Confocal micrographs of longitudinal sections of the proximal, medial and distal SI from infant mice treated with PBS or NAC after 24 h.
(PDF)

Video S1 **Visualization of** ***V. cholerae*** **in the distal SI by two-photon microscopy.** Video depicts VcGreen in an intact explant 24 h post orogastric inoculation. Green depicts VcGreen, white/yellow depicts intestinal villi autofluoresence. Images were acquired every 2 seconds. Video playback is at 15 frames per second. Scale bar, 10 um. Time displayed as hh:mm:ss.
(MOV)

Video S2 **Visualization of** ***V. cholerae*** **in the distal SI by two-photon microscopy.** Video depicts VcGreen in an intact explant 24 h post orogastric inoculation. Green depicts VcGreen, white/yellow depicts intestinal villi autofluoresence. Images were acquired every ∼1.5 seconds. Video playback is at 15 frames per second. Scale bar, 10 um. Time displayed as hh:mm:ss.
(MOV)
