# Supplementary figures and images for: Insights into Vibrio cholerae Intestinal Colonization from Monitoring Fluorescently Labeled Bacteria
Source: PLoS Pathog. 2014 Oct 2;10(10):e1004405. doi: 10.1371/journal.ppat.1004405 (PMC4183697; doi:10.1371/journal.ppat.1004405)

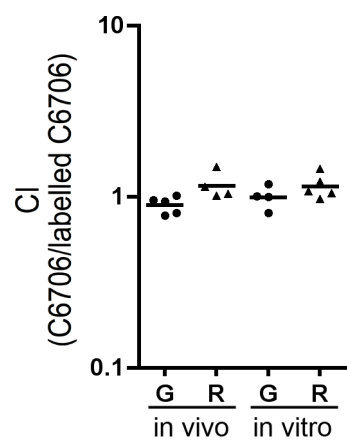

Fig. S1

Supplement: Figure S1 — VcGreen and VcRed exhibit WT growth in vivo and in vitro. Competition assays between VcGreen (G) or VcRed (R) vs the parental strain C6706 in the SI of infant mice (in vivo) and in vitro in LB. Bars represent the geometric mean. (PDF) [file ppat.1004405.s001.pdf]

A

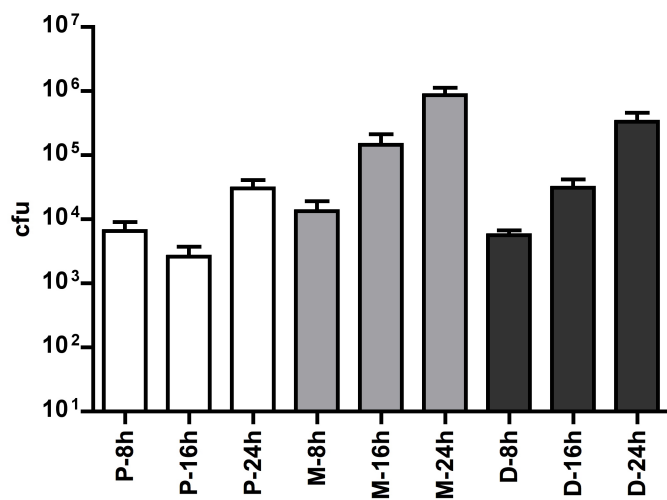

B

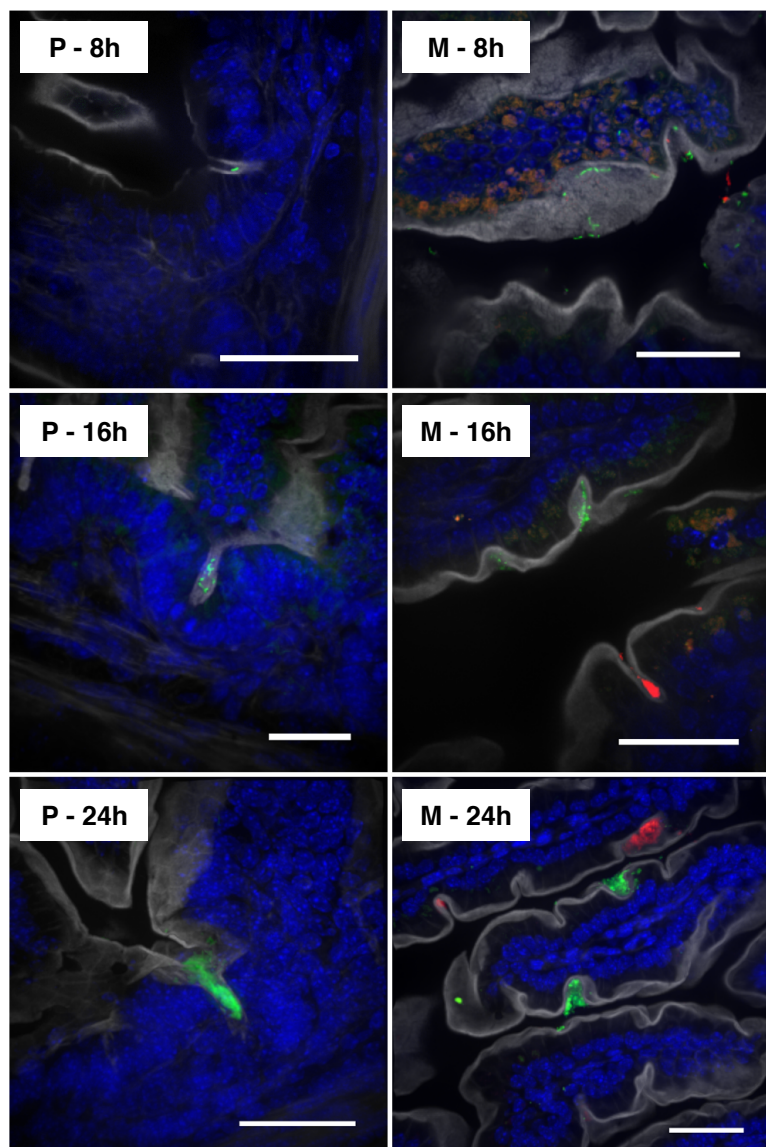

Fig. S2

Supplement: Figure S2 — Spatial and temporal differences in CFU recovered from the small intestine. The small intestines of infant mice co-inoculated with VcRed and VcGreen were divided into three equal parts and the central 1 cm segments of the proximal (P), medial (M) and distal (D) parts were used for plating and microscopic analyses. (A) Numbers of CFUs recovered from homogenates of each segment at 8, 16, or 24 hr PI. Mean values and SEM are plotted. (B) Confocal micrographs showing VcRed and VcGreen distribution in the proximal and medial segments at these time points. Tissue sections were counterstained with DAPI (blue) and phalloidin (gray). Scale bars = 50 µm. (PDF) [file ppat.1004405.s002.pdf]

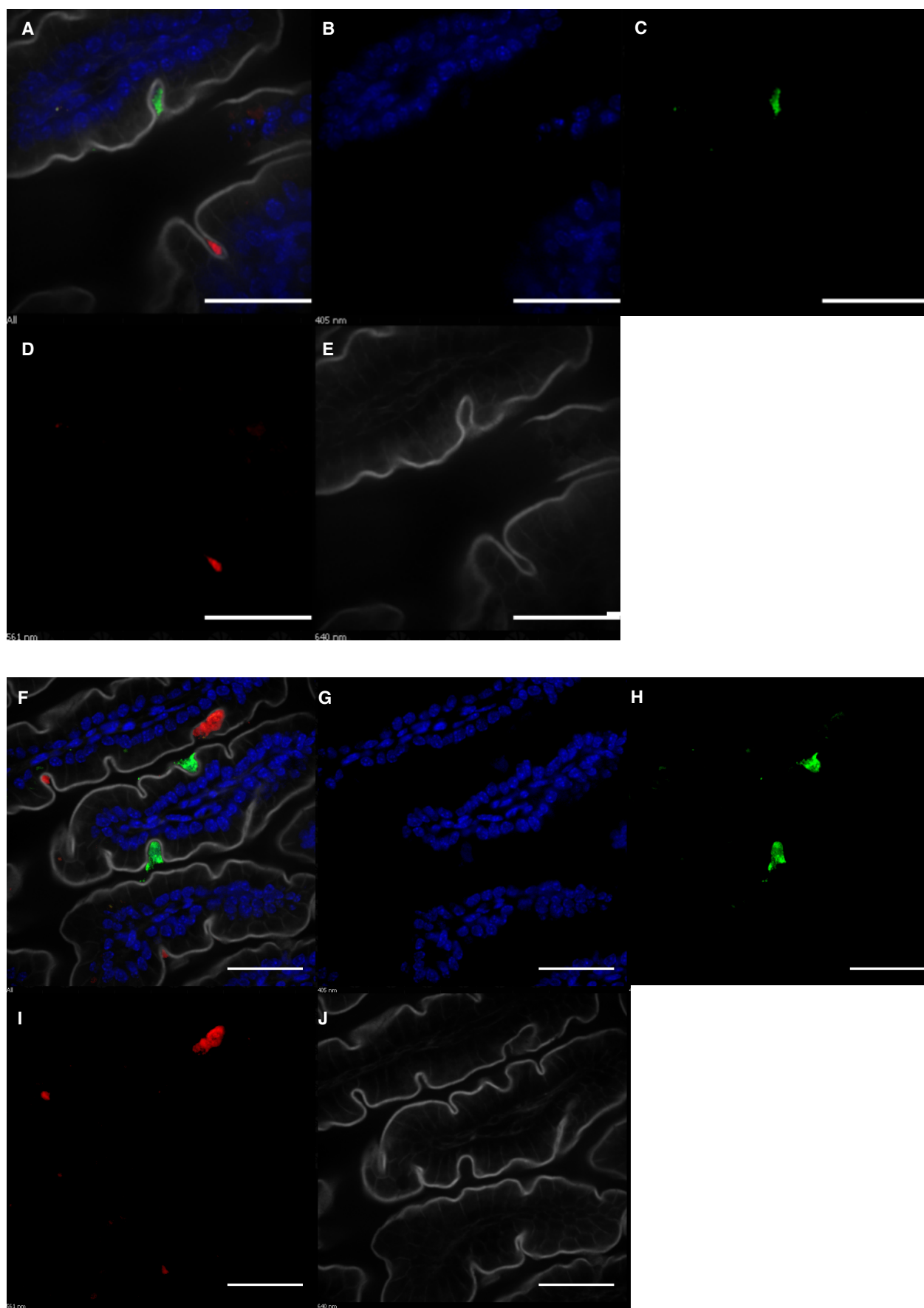

**Fig. S3**

Supplement: Figure S3 — Confocal micrographs of intraintestinal VcRed and VcGreen. Tissue from the medial small intestines of animals coinfected with VcRed and VcGreen for 24 hr was stained with DAPI (blue) and phalloidin (gray) Individual channels showing DAPI (B, G), VcGreen (C, H), VcRed (D, I), and phalloidin (E, J) are shown, as well as merged images (A, F). Scale bars, 50 µm. (PDF) [file ppat.1004405.s003.pdf]

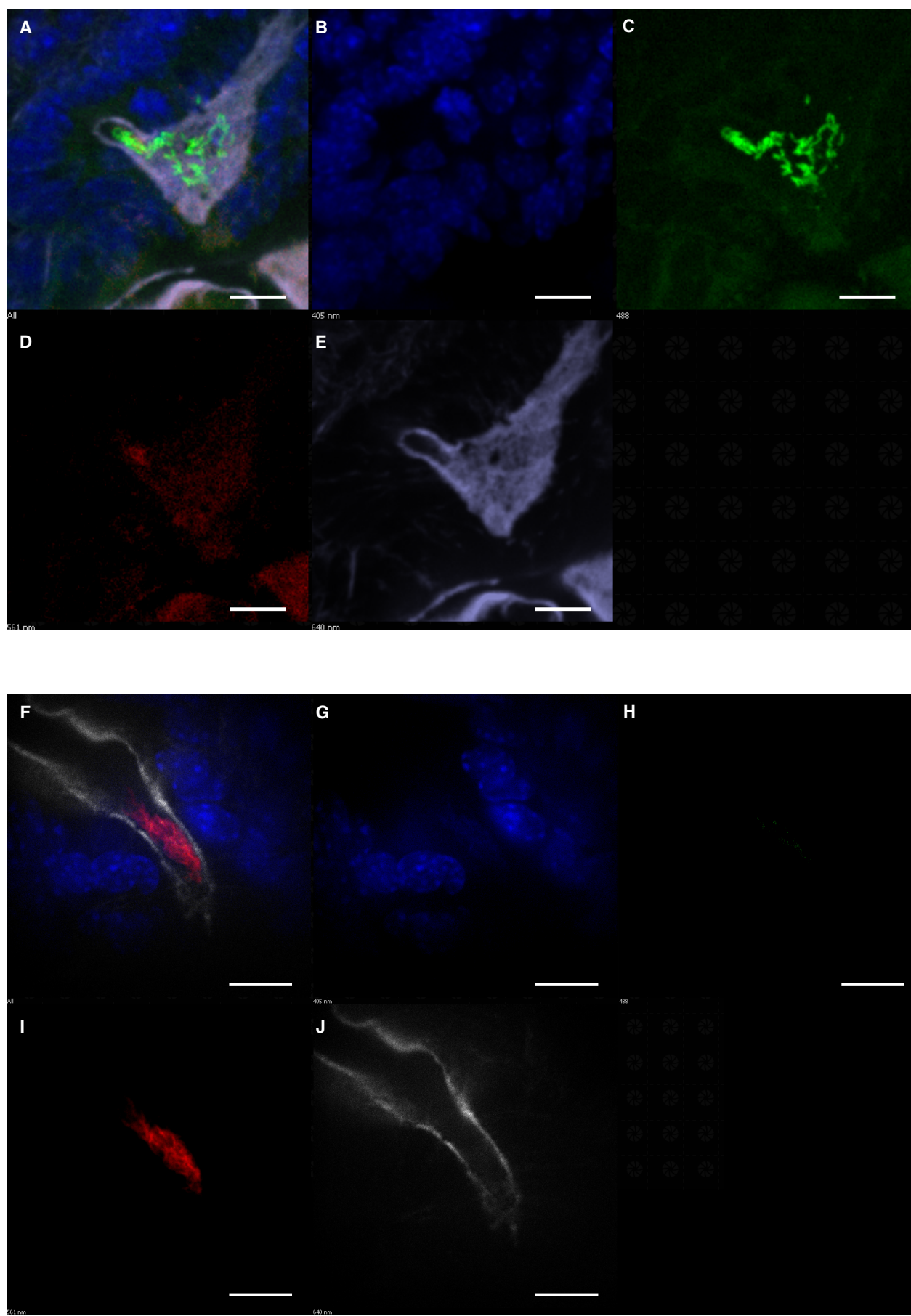

**Fig. S4**

Supplement: Figure S4 — High magnification confocal micrographs of intraintestinal VcRed and VcGreen, showing individual cells. Tissue from the small intestine of animals coinfected with VcRed and VcGreen for 24 hr was stained with DAPI (blue) and phalloidin (gray). Individual channels showing DAPI (B, G), VcGreen (C, H), VcRed (D, I), and phalloidin (E, J) are shown, as well as merged images (A, F). Scale bars, 10 µm (A–E) and 25 µm (F–J). (PDF) [file ppat.1004405.s004.pdf]

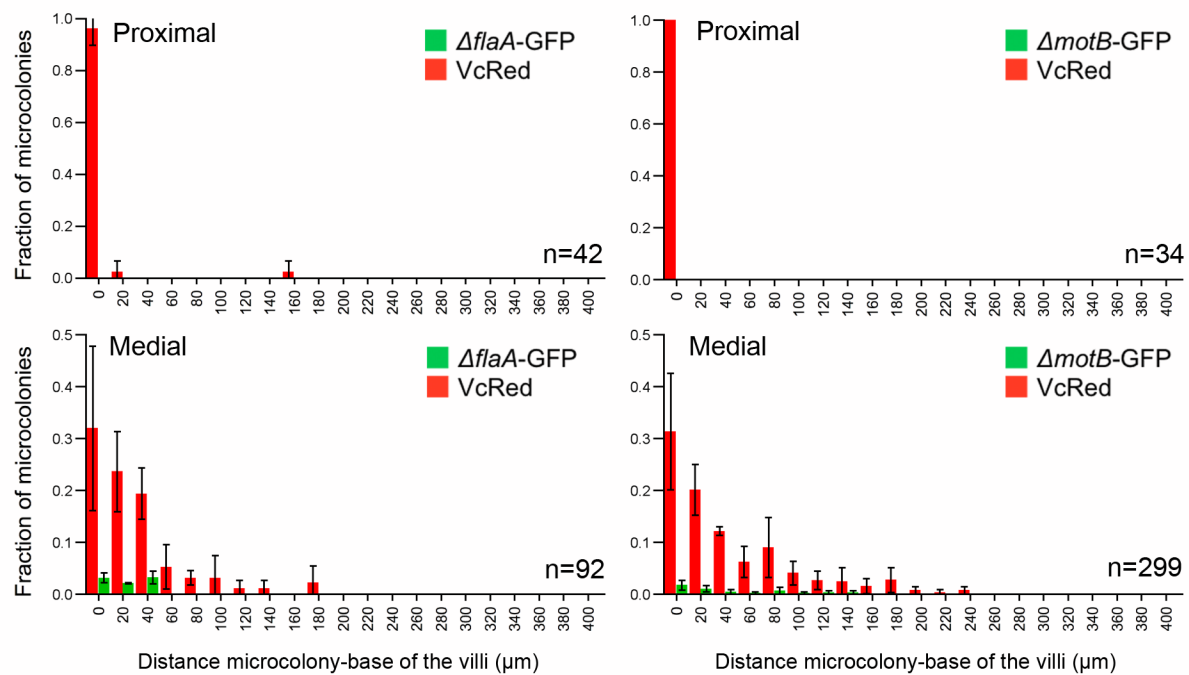

**Fig. S5**

Supplement: Figure S5 — Distribution of ΔflaA and ΔmotB microcolonies along the axes of intestinal villi in the proximal and distal SI segments. The distance separating microcolonies from the base of the villi was measured by confocal microscopy in tissue cross sections from three mice co-inoculated with GFP-labeled ΔflaA or ΔmotB and VcRed. Data represent the mean ±SD. The number (n) of microcolonies analyzed is indicated in the bottom right of each panel. (PDF) [file ppat.1004405.s005.pdf]

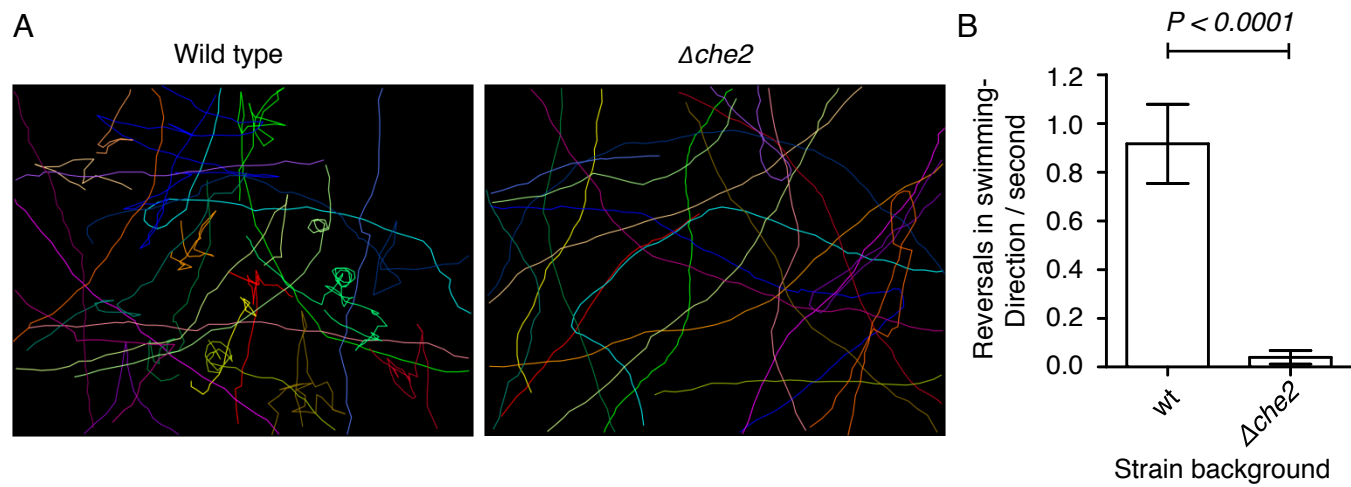

**Fig. S6**

Supplement: Figure S6 — The frequency of reversals in swimming direction of the Δche2 mutant is reduced compared with the wild type. Images of swimming wild type and Δche2 mutant cells (A) and reversal frequency/sec (B). Images of swimming cells were recorded and analyzed as previously described [41]. (PDF) [file ppat.1004405.s006.pdf]

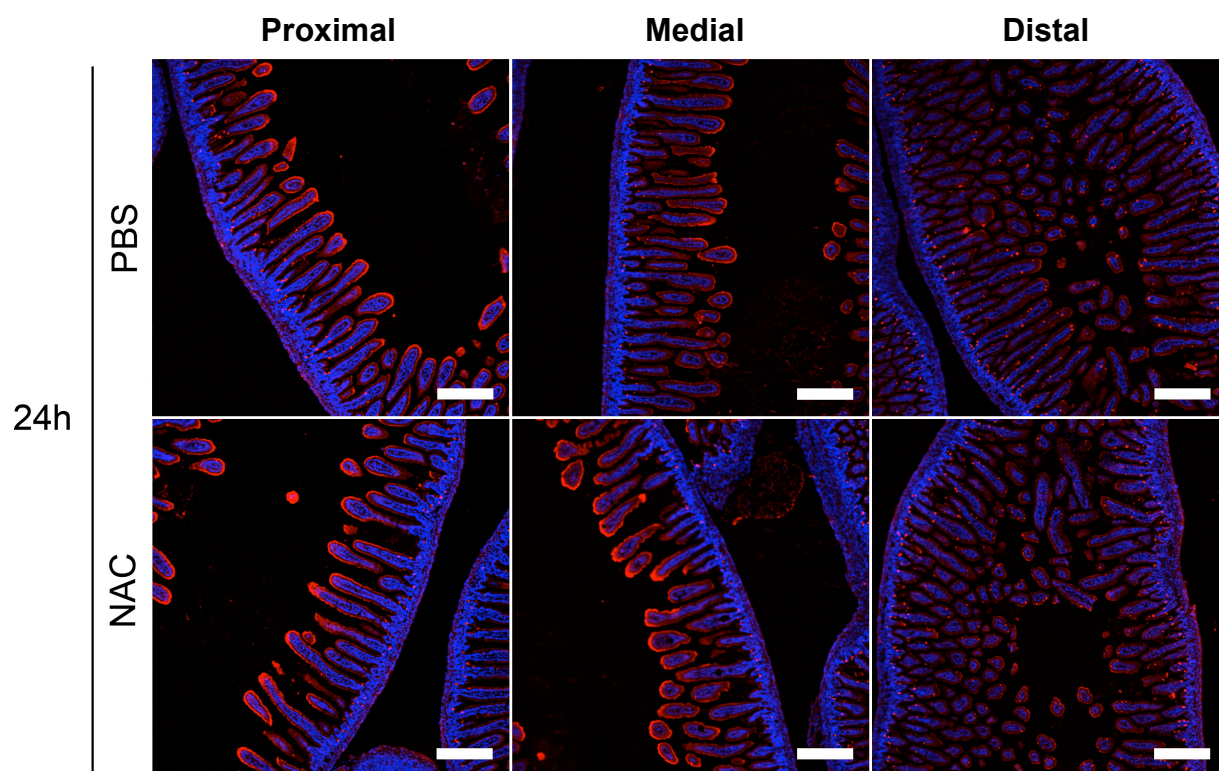

Fig. S7

Supplement: Figure S7 — The influence of NAC treatment is no longer detectable 24 h after treatment. Confocal micrographs of longitudinal sections of the proximal, medial and distal SI from infant mice treated with PBS or NAC after 24 h. (PDF) [file ppat.1004405.s007.pdf]
